# Warum brauchen wir Leitlinien für Suizidprävention?

**DOI:** 10.1007/s00103-021-03468-w

**Published:** 2021-12-30

**Authors:** Barbara Schneider, Andreas Reif, Birgit Wagner, Manfred Wolfersdorf

**Affiliations:** 1 Klinik für Psychiatrie, Psychosomatik und Psychotherapie, Johann Wolfgang-Goethe-Universität Frankfurt/Main, Frankfurt/Main, Deutschland; 2 Abteilung für Abhängigkeitserkrankungen, Psychiatrie und Psychotherapie, LVR-Klinik Köln, Wilhelm-Griesinger-Str. 23, 51109 Köln, Deutschland; 3 MSB Medical School Berlin, Berlin, Deutschland; 4 Universität Bayreuth, Bayreuth, Deutschland

**Keywords:** Suizidalität, Postvention, Leitlinien, Evidenzbasierung, Konsens, Suicidality, Postvention, Guidelines, Evidence-based, Consensus

## Abstract

Trotz der Relevanz des Themas Suizidalität und gut bekannter Risikofaktoren gibt es bisher keine deutsche Leitlinie zur Suizidalität im Erwachsenenalter. In diesem Beitrag werden zunächst die Geschichte und die Hintergründe der Arbeit mit Leitlinien beschrieben. Der aktuelle Stand der Leitlinien für psychische Erkrankungen in Deutschland wird dargestellt und auf suizidpräventive Inhalte hin untersucht. Die Notwendigkeit evidenzbasierter Suizidprävention und einer spezifischen Leitlinie zur Suizidprävention bei Erwachsenen wird diskutiert.

Nur durch gezielte Suizidpräventionsstrategien und Interventionen für die jeweiligen Risikogruppen und unter Beachtung von Alters- und Geschlechtsspezifität kann für alle Betroffenen eine flächendeckende, gut erreichbare, bedarfs- und versorgungsgerechte, finanzierbare sowie nachhaltige medizinische Versorgung auf einem hohen Niveau sichergestellt werden. Dies gilt für den ambulanten und den stationären Bereich sowie für deren Schnittstellen. Bei Suizidalität handelt es sich um ein diagnoseübergreifendes, in unterschiedlichen Versorgungskontexten auftretendes Syndrom mit komplexem Behandlungsbedarf, weshalb intersektorale und multiprofessionelle Aspekte in einer entsprechenden Leitlinie besonders zu adressieren sind. Wissenschaftliche Evidenz und interdisziplinärer Konsens unter Expertinnen und Experten zum Umgang mit suizidalem Verhalten in der medizinischen Versorgung können dazu beitragen, Morbidität und Mortalität im Zusammenhang mit Suizidalität zu reduzieren. Im August 2021 wurde die Finanzierung einer S3-Leitlinie „Umgang mit Suizidalität“ vom Innovationsfonds des Gemeinsamen Bundesausschusses bewilligt.

## Abstract (en)

At present, there are no German guidelines regarding suicidal behaviors in adulthood despite their relevance to public health and the well-established evidence about their prevention. This paper first describes the history and background of working with guidelines. The current status of guidelines for mental illness in Germany is presented and examined for suicide-preventive content. The need for evidence-based suicide prevention and a specific guideline for suicide prevention in adults is discussed.

Only via targeted suicide prevention strategies and interventions for the respective risk groups, and by paying particular attention to age and gender specificity in the outpatient as well as inpatient sector, can a high level of care for all patients be ensured. Such strategies have to pay specific attention to the interface between the individual care sectors and need to take comprehensive, easily accessible, needs-based, and affordable sustainable medical care into account. This applies to the outpatient and inpatient sectors as well as to their interfaces. Suicidality is a cross-diagnosis syndrome that occurs in different care contexts and requires complex treatment; therefore, intersectoral and multiprofessional aspects must particularly be addressed in the guideline. Scientific evidence and interdisciplinary expert consensus on the management of suicidal behavior in medical care can help reduce morbidity and mortality associated with suicidality. In August 2021, the funding of an S3 guideline “Management of Suicidality” was approved by the Federal Joint Committee.

## Einleitung

In Deutschland nehmen sich jährlich mehr als 9000 Menschen das Leben (2019: 9041 Suizide – davon 6842 Männer und 2199 Frauen [1] – entsprechend einer Suizidrate von 11,6/100.000 Einwohner). In Mitteleuropa steigt mit zunehmendem Lebensalter die Suizidrate an, insbesondere bei den Männern. Es wird davon ausgegangen, dass jeder Suizid im Mittel 8–10 weitere Menschen unmittelbar in Mitleidenschaft zieht [2], also pro Jahr weitere ca. 70.000–90.000. Bei jüngeren Menschen ist Suizid eine der häufigsten Todesursachen. Es gibt bereits in der 4. Überarbeitung eine deutsche Leitlinie der Arbeitsgemeinschaft der Wissenschaftlichen Medizinischen Fachgesellschaften (AWMF) zum Thema Suizidalität im Kindes- und Jugendalter [3], die unter Federführung der Deutschen Gesellschaft für Kinder- und Jugendpsychiatrie, Psychosomatik und Psychotherapie e. V. (DGKJP) entstanden ist. Derzeit steht die fünfte Aktualisierung dieser Leitlinie an. Eine Leitlinie zum Umgang mit Suizidalität im Erwachsenenalter gibt es bisher nicht. Im August 2021 wurde jedoch die Finanzierung einer S3-Leitlinie „Umgang mit Suizidalität“ vom Innovationsfonds des Gemeinsamen Bundesausschusses bewilligt.

In Deutschland unterstützt und fördert die AWMF die Entwicklung von Leitlinien u. a. zur Behandlung von psychischen und somatischen Erkrankungen. Im Jahr 1995 hat der Sachverständigenrat für die Konzertierte Aktion im Gesundheitswesen die AWMF erstmalig beauftragt, die Entwicklung von Standards, Richtlinien, Leitlinien und Empfehlungen der wissenschaftlichen medizinischen Fachgesellschaften zu koordinieren. Inzwischen sind in Deutschland insgesamt mehr als 800 Leitlinien erschienen. Es besteht jedoch bisher keine dezidierte deutschsprachige Leitlinie für den Umgang mit Suizidalität bei Erwachsenen, obwohl suizidales Verhalten ein wesentlicher Faktor von Mortalität und Morbidität ist und auch ein großes gesellschaftliches Problem darstellt.

Im Vereinigten Königreich existiert die englischsprachige (evidenzbasierte) Leitlinie des National Institute for Health and Care Excellence (NICE-Guideline) „Suicide prevention“ (letzte Überarbeitung 2018 [4]). Aufgrund der unterschiedlichen Versorgungssysteme lässt sie sich jedoch nur schwer auf die deutsche Situation anwenden und übertragen. Darüber hinaus unterscheidet sich die zugrunde liegende NICE-Methodologie grundsätzlich von der AWMF-S3-Methodik, die sowohl evidenzbasiert als auch konsensorientiert ist [5, 6]. Auch außerhalb der psychiatrisch-psychotherapeutischen Versorgung hat suizidales Verhalten eine hohe Relevanz, in der hausärztlichen Versorgung, aber vor allem in der Neurologie, der Chirurgie, der Allgemeinmedizin/Inneren Medizin einschließlich der Geriatrie. Insbesondere muss im Zusammenhang mit onkologischen und palliativmedizinischen Fragestellungen an Suizidalität gedacht werden. Suizidversuche führen häufig zur Inanspruchnahme des Rettungsdienstes, der Notaufnahmen und zu intensivmedizinischer Versorgung. Die entsprechenden (Weiter‑)Behandlungsketten, z. B. Vermittlung von fachpsychiatrischer Evaluation einer zugrunde liegenden psychischen Erkrankung und spezifische Behandlung des suizidalen Syndroms, sind jedoch nicht standardisiert und es kommt sowohl (häufig) zu Unter- als auch (seltener) zu Überversorgung. Die Qualifikation im Hinblick auf den Umgang mit suizidalem Verhalten ist sehr heterogen; da bislang keine Leitlinie existiert, besteht hier häufig Unklarheit im Hinblick auf das weitere Vorgehen und somit eine erhebliche Versorgungslücke.

## Grundbegriffe und epidemiologische Grundlagen

Es gibt eine Vielzahl von Definitionen von Suizidalität. Die Weltgesundheitsorganisation (WHO; [7]) wählte eine pragmatische Definition, die Suizidgedanken, Suizidabsichten, Suizidpläne, Suizidversuche und Suizide umfasst. Unter dem Begriff „Suizidalität“ werden somit alle Gefühle, Gedanken, Impulse und Handlungen zusammengefasst, die selbstzerstörerischen Charakter haben und das eigene Versterben aktiv oder durch Unterlassung anstreben bzw. direkt oder indirekt in Kauf nehmen. Im wissenschaftlichen Sprachgebrauch wird grundsätzlich der wertfreie Begriff „Suizid“ und nicht die wertenden Begriffe „Selbstmord“ oder „Freitod“ verwendet. Aktuelle, umfassende Übersichtsarbeiten zum Thema Suizidalität wurden in den letzten Jahren von Turecki und Brent [8] und Fazel und Runeson [9] vorgelegt.

Suizidversuche als solche werden im Gegensatz zu vollendeten Suiziden in Deutschland nicht systematisch erfasst, weisen eine hohe Dunkelziffer auf und können lediglich durch Stichprobenhochrechnungen ermittelt werden. Nach Einschätzung der WHO [7] ereignen sich jährlich 10- bis 30-mal so viele Suizidversuche wie Suizide. Demnach kann man in Deutschland von geschätzt 100.000 bis hin zu 300.000 Suizidversuchen ausgehen. Eine signifikante Anzahl hiervon (ca. 20–30 %) führt zur Inanspruchnahme von medizinischen Versorgungsleistungen unterschiedlicher Art [1]. Die fehlende Erfassung von Suizidversuchen ist daher ein Problem, weil vorangegangene Suizidversuche einen der wichtigsten Risikofaktoren für den vollendeten Suizid darstellen [10, 11], sodass die Chance zur individuellen Sekundärprävention vertan wird. Aber auch säkulare Trends im Hinblick auf Methoden oder Orte werden deshalb übersehen, was präventives Eingreifen erschwert.

In Mitteleuropa steigt mit zunehmendem Lebensalter die Suizidrate an, insbesondere jedoch bei den Männern [1]. Die Suizidprävention im Alter ist eine besondere Herausforderung, was durch den demografischen Wandel in den kommenden Jahren weiter verstärkt wird. Hinzu kommt, dass in Deutschland eine Neuregelung eines Gesetzes zum assistierten Suizid nach dem Urteil des Bundesverfassungsgerichts (BVerfG) vom 26.02.2020 ansteht [12]; in fast allen Ländern mit einer liberalen Gesetzgebung zum assistierten Suizid steigen die Zahlen der eigenständigen Suizide an, (z. B. [13], siehe [14]), sodass davon ausgegangen werden muss, dass auch in Deutschland die Zahl der Suizide zunimmt, falls eine Liberalisierung der Gesetzgebung zum assistierten Suizid eintreten würde.

Meist, aber nicht immer liegt bei einem Suizid eine schwere psychische Erkrankung vor; hinzu kommen meist endogen oder exogen getriggerte, krisenhafte Zuspitzungen als hinreichende Faktoren. Suizidalität ist für das Individuum (und seine Angehörigen) sehr belastend und hat negative psychische, soziale und körperliche Folgen. Suizidalität entsteht im Zusammenspiel von individuell-biografischen, somatisch-gesundheitlichen und gesellschaftlich-kulturellen Einflussfaktoren in unterschiedlicher Gewichtung sowie aktuellen Lebensereignissen und ist somit ein komplexes Phänomen. Suizidalität als Phänomen ist meist vorübergehend, Suizid jedoch endgültig, was die Notwendigkeit von adäquatem, evidenzbasiertem Umgang mit Suizidalität aufzeigt.

Mit dem höchsten Suizidrisiko ist das Vorliegen einer psychischen Erkrankung assoziiert – bei bis zu 90 % aller vollendeten Suizide lässt sich eine solche prämortal nachweisen –, jedoch kann dennoch nicht automatisch von Suizidalität auf das Vorliegen einer psychischen Erkrankung geschlossen werden [15]; selbst bei einem vollendeten Suizid liegt insbesondere bei jungen Männern oft keine psychiatrische Diagnose vor [16].

Bei beinahe allen psychischen Erkrankungen ist das Suizidrisiko deutlich erhöht [17], insbesondere jedoch bei affektiven Erkrankungen (Depression, bipolare Störung), Alkohol- und Drogenabhängigkeit, schizophrenen Psychosen, Persönlichkeitsstörungen und auch Schlafstörungen [18–21]. Die vorliegenden Metaanalysen sowie auch neuere Untersuchungen zeigen, dass an affektiven Störungen Erkrankte ein etwa 20-fach erhöhtes Suizidrisiko im Vergleich zur restlichen Bevölkerung haben [18, 19, 22]. Bei Personen mit Alkoholmissbrauch und -abhängigkeit besteht ein fast 6‑fach erhöhtes Suizidrisiko, wobei das Suizidrisiko bei Frauen stärker erhöht ist [20]. Patientinnen und Patienten, die an mehr als einer psychischen Erkrankung (Komorbidität) leiden, haben ein besonders stark erhöhtes Suizidrisiko [23].

Körperliche Erkrankungen wie bösartige Tumorerkrankungen, Niereninsuffizienz, Schlaganfall und andere neurologische Erkrankungen sind ebenfalls Risikofaktoren für Suizid [24–28]. Zudem sind verschiedene soziodemografische Faktoren wie Unverheiratetsein, Alleineleben und LGBTQI+ (lesbisch, schwul, bisexuell, trans, queer, intersexuell und weitere Geschlechtsidentitäten) Risikofaktoren für Suizid [29–31]. Auch Migration ist mit Suizidalität assoziiert, insbesondere mit Suizidversuchen [32]. Als ein weiterer Risikofaktor gilt Suizidalität in der Familienanamnese [33].

## Hintergrund: Geschichte der Leitlinienentwicklung in Deutschland

In Deutschland sind in der AWMF 180 wissenschaftliche Fachgesellschaften aus allen Bereichen der Medizin zusammengeschlossen. Die AWMF hat das AWMF-Regelwerk zur Erstellung und Publikation aktueller und hochwertiger Leitlinien der wissenschaftlichen medizinischen Fachgesellschaften im AWMF-Leitlinienregister erstellt. Darüber hinaus gibt es seit 2003 die Initiative der Nationalen Versorgungsleitlinien (NVL), an der neben der AWMF auch die Bundesärztekammer und die Kassenärztliche Bundesvereinigung beteiligt sind. Die NVL wird obligat vom Ärztlichen Zentrum für Qualität in der Medizin (ÄZQ) und nicht von Fachgesellschaften koordiniert. Die einzige NVL im Bereich der Psychiatrie betrifft das Krankheitsbild der unipolaren Depression. Im Unterschied zu den Leitlinien, die unter Federführung der AWMF erstellt sind, beinhalten die Nationalen Versorgungsleitlinien auch *immer* eine Patientenleitlinie, welche sich direkt an Patientinnen und Patienten richtet.

Leitlinien stellen den nach einem definierten, transparent gemachten Vorgehen erzielten Konsens mehrerer Expertinnen und Experten aus unterschiedlichen Fachbereichen und Arbeitsgruppen (möglichst unter Einbeziehung von anderen Fachberufen des Gesundheitswesens und Patientinnen und Patienten) zu medizinischen Vorgehensweisen dar. Der Konsens richtet sich nach der wissenschaftlichen Evidenz, wobei Metaanalysen u. Ä. die größte Bedeutung zukommt. Lediglich für Bereiche, bei denen es keinerlei kontrollierte Studien gibt, wird ein klinischer Konsenspunkt formuliert. Leitlinien richten sich vor allem an Ärztinnen und Ärzte, aber auch an Pflegekräfte, psychologische Psychotherapeutinnen und Psychotherapeuten und andere Fachleute im Gesundheitswesen.

## Warum arbeiten wir mit Leitlinien?

Leitlinien sind systematisch entwickelte, wissenschaftlich begründete und praxisorientierte Entscheidungshilfen für die angemessene medizinische Vorgehensweise bei gesundheitlichen Problemen. Vorrangiges Ziel von Leitlinien ist die Verbesserung der Qualität medizinischer Versorgung durch Wissensvermittlung auf dem neuesten Stand der Wissenschaft im Sinn der evidenzbasierten Medizin. Einerseits werden Empfehlungen abgegeben für Maßnahmen, die evidenzbasiert und unter Abwägung des Risiko-Nutzen-Profils effektiv sind; andererseits wird von Maßnahmen abgeraten, für die in Studien kein Wirksamkeitsnachweis geführt werden konnte oder bei denen Nichtwirksamkeit oder sogar ein negativer Effekt nachgewiesen wurde. Leitlinien haben die Aufgabe, die wissenschaftliche Evidenz zu speziellen medizinischen Fragestellungen auf dem Boden der klinischen Erfahrung explizit darzulegen, unter methodischen und klinischen Aspekten zu bewerten, gegensätzliche Standpunkte zu klären sowie unter Abwägung von Nutzen und Schaden das derzeitige Vorgehen der Wahl zu definieren. Im Gegensatz zu Richtlinien sind sie rechtlich nicht verbindlich. Das heißt, Ärztinnen und Ärzte und psychologische Psychotherapeutinnen und Psychotherapeuten können von der in der Leitlinie empfohlenen Behandlung abweichen, wenn sie denken, dass die dargestellten Empfehlungen für bestimmte Patientinnen und Patienten nicht geeignet sind. Abweichungen sollten aber jeweils begründet sein. Leitlinien werden regelmäßig aktualisiert; aktuell diskutiert wird eine laufende Aktualisierung im Sinne einer „living guideline“, was insbesondere auf Gebieten mit sich rasch ändernden Empfehlungen (bspw. Onkologie) sehr sinnvoll scheint. Hierbei sind digitale Lösungen, wie beispielsweise „MAGICapp“^1^ hilfreich und werden zukünftig zunehmend eingesetzt.

Die Evidenzbasierung ist maßgebend für die *wissenschaftliche Legitimation* einer Leitlinie, die repräsentative Beteiligung der Anwenderinnen und Anwender sowie die strukturierte Konsensfindung sind dagegen entscheidend für die *Akzeptanz und Umsetzung*. Auf der Basis von Charakteristika bezüglich der Konsensfindung und systematischer Literaturrecherche werden nach der AWMF 4 Klassen von Leitlinien eingeteilt: S1 (Handlungsempfehlungen von Expertinnen und Experten), S2k (konsensbasiert), S2e (evidenzbasiert) und S3 (evidenz- und konsensbasiert; [34]).

## Aktueller Stand der Leitlinien für psychische Erkrankungen in Deutschland

In Deutschland gibt es eine Leitlinie zur Suizidalität bei Kindern und Jugendlichen, die derzeit noch nicht aktualisiert ist. Des Weiteren gibt es aktuell eine abgelaufene klinische Leitlinie zu nichtsuizidalem, selbstverletzendem Verhalten (NSSV) bei Kindern und Jugendlichen (Tab. 1), die eine sinnvolle Ergänzung zur Leitlinie „Suizidalität im Kindes- und Jugendalter“ [3] ist. Zudem ist eine Abgrenzung von NSSV und akuter Suizidalität wichtig und sinnvoll. Zur Suizidalität und zur Suizidprävention im Erwachsenenalter existiert bisher in Deutschland keine eigene Leitlinie. Somit gibt es derzeit keine einzige gültige Leitlinie bezüglich des Umgangs mit Suizidalität und zur Suizidprävention in Deutschland.

**Table 1:** 

Leitlinien unter DGPPN und/oder DGKJP-Federführung	AWMF-Registernummer	Stufe	Status	Suizidalität erwähnt
*Berücksichtigung der Suizidalität auf jeden Fall erforderlich*				
Aufmerksamkeitsdefizit-Hyperaktivitätsstörung (ADHS) bei Kindern, Jugendlichen und Erwachsenen	028-045	S3	Gültig	3‑mal erwähnt
Autismus-Spektrum-Störungen im Kindes‑, Jugend- und Erwachsenenalter, Teil 1: Diagnostik	028-018	S3	Gültig	3‑mal erwähnt (u. a. als Aspekt, den die Anamneseerhebung beinhalten soll)
Autismus-Spektrum-Störungen im Kindes‑, Jugend- und Erwachsenenalter, Teil 2: Therapie	028-047	S3	Gültig	Empfehlung 90: C.7.4.12 Umgang mit Suizidalität (konsensbasiert)
Begutachtung psychischer und psychosomatischer Erkrankungen	051-029	S2k	Gültig	2‑mal erwähnt
Bipolare Störungen	038-019	S3	Gültig	Deutsche Gesellschaft für Suizidprävention ist beteiligt
				Gesondertes ausführliches Kapitel hierzu; ein Empfehlungsgrad A und ein Empfehlungsgrad B
				14 Empfehlungen zur Suizidalität
Demenzen	038-013	S3	Gültig	–
Diagnostik und Behandlung von Zwangsstörungen im Kindes- und Jugendalter	028-007	S3	Gültig	10-mal erwähnt
Einwilligung bei Demenzen	108-001	S2k	Gültig	Erscheint nur im Literaturverzeichnis
Essstörungen, Diagnostik und Therapie	051-026	S3	Gültig	22-mal erwähnt
Intelligenzminderung	028-042	S2k	Gültig	2‑mal erwähnt
Medikamentenbezogene Störungen	038-025	S3	Gültig	8‑mal erwähnt
Metamphetamin bezogene Störungen	038-024	S3	Gültig	10-mal erwähnt
Nichtorganische Schlafstörungen (F51)	028-012	S1	Gültig	2‑mal erwähnt
Notfallpsychiatrie	038-023	S2k	Gültig	Eigenes Kapitel über Suizid
Psychosoziale Therapien bei schweren psychischen Erkrankungen	038-020	S3	Gültig	20-mal erwähnt
Rauchen und Tabakabhängigkeit: Screening, Diagnostik und Behandlung	076-006	S3	Gültig	11-mal erwähnt
Schizophrenie	038-009	S3	Gültig	Eigenes Kapitel über Suizidalität
Screening, Diagnose und Behandlung alkoholbezogener Störungen	076-001	S3	Gültig	12-mal erwähnt
Verhinderung von Zwang: Prävention und Therapie aggressiven Verhaltens bei Erwachsenen	038-022	S3	Gültig	11-mal erwähnt
Kindesmisshandlung, -missbrauch, -vernachlässigung unter Einbindung der Jugendhilfe und Pädagogik (Kinderschutzleitlinie)	027-069	S3	Gültig	5‑mal erwähnt
Behandlung von depressiven Störungen bei Kindern und Jugendlichen	028-043	S3	In Überarbeitung	48-mal erwähnt
Enuresis und nichtorganische (funktionelle) Harninkontinenz bei Kindern und Jugendlichen	028-026	S2k	In Überarbeitung	–
Zwangsstörungen	038-017	S3	In Überarbeitung	3‑mal erwähnt
Suizidalität im Kindes- und Jugendalter	028-031	S2k	Anmeldeverfahren für Überarbeitung noch nicht abgeschlossen	Eigene Leitlinie für Suizidalität
Nichtsuizidales Selbstverletzendes Verhalten (NSSV) im Kindes- und Jugendalter	028-029	S2k	Abgelaufen	Leitlinie dient zur Abgrenzung gegenüber Suizidalität
Autonomieförderung und Prävention von Zwangsmaßnahmen, Unterbringungen und freiheitsentziehenden Maßnahmen in der kinder- und jugendpsychiatrischen und -psychotherapeutischen Behandlung	028-048	S2k	Angemeldet	N. z.
Behandlung Cannabis bezogener Störungen	076-005	S3	Angemeldet	N. z.
Geschlechtsinkongruenz und Geschlechtsdysphorie im Kindes- und Jugendalter: Diagnostik und Behandlung	028-014	S3	Angemeldet	N. z.
Umgang mit Suizidalität	038-028	S3	Angemeldet	Eigene Leitlinie für Suizidalität
*„Suizidalität“ könnte ggf. berücksichtigt werden*				
Diagnostik und Behandlung der Rechenstörung	028-046	S3	Gültig	–
Gutachtliche Untersuchung bei psychischen und psychosomatischen Störungen	051-029	S2k	Gültig	2‑mal erwähnt
Störungen des Sozialverhaltens: Empfehlungen zur Versorgung und Behandlung	028-020	S3	Gültig bis 09/21	2‑mal erwähnt
Diagnostik und Behandlung bei der Lese- und/oder Rechtschreibstörung	028-044	S3	In Überarbeitung	–
Sprachentwicklungsstörungen (SES) unter Berücksichtigung umschriebener Sprachentwicklungsstörungen (USES), Diagnostik	049-006	S2k	Überarbeitung für 2021 geplant	–

*AWMF* Arbeitsgemeinschaft der Wissenschaftlichen Medizinischen Fachgesellschaften; *DGKJP* Deutsche Gesellschaft für Kinder- und Jugendpsychiatrie, Psychosomatik und Psychotherapie e. V.; *DGPPN* Deutsche Gesellschaft für Psychiatrie, Psychotherapie, Psychosomatik und Nervenheilkunde e. V.; *N. z.* Nicht zutreffend; *S1* Handlungsempfehlungen von Expertinnen und Experten, *S2k* konsensbasiert, *S2e* evidenzbasiert, *S3* evidenz- und konsensbasiert

Zwar sind in den vergangenen Jahren unter der Federführung der DGPPN und der DGKJP eine Reihe von Leitlinien zu psychischen Erkrankungen erschienen (Tab. 1) oder auch mit deren Beteiligung (Tab. 2; siehe auch [35]). Einige dieser Leitlinien haben eigene Kapitel zum Thema suizidales Verhalten. Auffällig ist, dass bei sehr vielen Leitlinien das Wort „Suizid“ bzw. dessen Ableitungen oder Synonyme nicht einmal erwähnt wurden. „Suizidalität“ müsste bzw. sollte nach Ansicht der Autorinnen und Autoren jedoch in den meisten der in den Tab. 1 und 2 aufgeführten Leitlinien stärker berücksichtigt werden, da die jeweils darin bearbeiteten Störungen nachgewiesene Risikofaktoren für Suizid sind oder – wie aus der klinischen Praxis bekannt ist – oft zusammen mit Erkrankungen vorkommen, die Risikofaktoren für Suizid sind, z. B. Zahnbehandlungsangst bei Abhängigkeitserkrankungen. In diesen Leitlinien sollten zumindest entsprechende Hinweise und Verweise auf die Leitlinien zur „Suizidalität“ erfolgen – auch in den Leitlinien, die sich bereits sehr ausführlich der Thematik Suizidalität widmen, wie beispielsweise die Leitlinie zu Autismusspektrumstörungen.

**Table 2:** 

Leitlinien mit DGPPN- und/oder DGKJP-Beteiligung	AWMF-Registernummer	Stufe	Status	Suizidalität erwähnt
*Berücksichtigung der Suizidalität auf jeden Fall erforderlich*				
Ärztliche Begutachtung von Menschen mit chronischen Schmerzen	094-003	S3	Gültig	–
Akute Folgen psychischer Traumatisierung – Diagnostik und Behandlung	051-027	S2k	Gültig	8‑mal erwähnt
Angststörungen	051-028	S3	Gültig	34-mal erwähnt
Chorea/Morbus Huntington	030-028	S2k	Gültig	3‑mal erwähnt
Depression bei Menschen mit Querschnittslähmung: Besonderheiten in der Diagnostik und Behandlung	179-003	S1	Gültig	5‑mal erwähnt
Diagnostik, Therapie und Nachsorge des Hodgkin-Lymphoms bei erwachsenen Patienten	018-029OL	S3	Gültig	–
Fibromyalgiesyndrom: Definition, Pathophysiologie, Diagnostik und Therapie	145-004	S3	Gültig	8‑mal erwähnt, meist als „Nebenwirkung“ von Medikamenten
Funktionelle Körperbeschwerden	051-001	S3	Gültig	Exkurs zu: Suizidalität bei funktionellen und somatoformen Körperbeschwerden
Geschlechtsinkongruenz, Geschlechtsdysphorie: Diagnostik, Beratung und Behandlung	138-001	S3	Gültig	12-mal erwähnt
LONTS: Opioide, Langzeitanwendung zur Behandlung bei nicht tumorbedingten Schmerzen	145-003	S3	Gültig	3‑mal erwähnt
Nicht erholsamer Schlaf/Schlafstörungen – Schlafbezogene Atmungsstörungen	063-001	S3	Gültig	–
Palliativmedizin für Menschen mit einer nicht heilbaren Krebserkrankung	128-001OL	S3	Gültig	Kapitel über Todeswunsch und Suizidalität
Posttraumatische Belastungsstörung	155-001	S3	Gültig	20-mal erwähnt
Schmerzassessment bei älteren Menschen in der vollstationären Altenhilfe	145-001	S3	Gültig	Einmal erwähnt
Visuelle Wahrnehmungsstörungen	022-020	S2k	Gültig	–
Zahnbehandlungsangst bei Erwachsenen	083-020	S3	Gültig	–
Depression (Nationale Versorgungsleitlinie, NVL)	Nvl-005	S3	In Überarbeitung, Revision für 2022 erwartet	Separates Kapitel „Management bei Suizidgefahr“
Diagnostik, Therapie und Verlaufskontrolle des Diabetes mellitus im Kindes- und Jugendalter	057-016	S3	In Überarbeitung	–
Parkinson-Syndrom, idiopathisch	030-010	S3	In Überarbeitung	2‑mal erwähnt
Psychoonkologische Diagnostik, Beratung und Behandlung von erwachsenen Krebspatienten	032-051OL	S3	In Überarbeitung	7‑mal erwähnt
Opioidbezogene Störungen	076-012	S3	Angemeldet	N. z.
*„Suizidalität“ könnte ggf. berücksichtigt werden*				
Allgemeine Grundlagen der medizinischen Begutachtung	094-001	S2k	Gültig	–
Fertilitätserhalt bei onkologischen Erkrankungen	015-082	S2k	Gültig	–
Gesundheitliche Aspekte und Gestaltung von Nacht- und Schichtarbeit	002-030	S2k	Gültig	–
Hausärztliche Multimedikation	053-043	S3	Gültig	–
Hypersalivation	017-075	S2k	Gültig	–
Müdigkeit	053-002	S3	Gültig	–
Neuroborreliose	030-071	S3	Gültig	–
Operative Behandlung der distalen, mittleren und proximalen Hypospadie	006-026	S2k	Gültig	–
Peri- und Postmenopause – Diagnostik und Interventionen	015-062	S3	Gültig	–
Therapie und Prävention der Adipositas im Kindes- und Jugendalter	050-002	S3	Gültig	–
Down-Syndrom im Kindes- und Jugendalter	027-051	S2k	Gültig bis 07/21	–
Redeflussstörungen, Pathogenese, Diagnostik und Behandlung	049-013	S3	Gültig bis 08/21	–
Chronischer Tinnitus	017-064	S3	In Überarbeitung	–
Depersonalisations-Derealisationssyndrom, Diagnostik und Behandlung	051-030	S2k	In Überarbeitung	–
Epidurale Rückenmarksstimulation zur Therapie chronischer Schmerzen	008-023	S3	In Überarbeitung	–
Transition von jungen Menschen mit Adipositas von der Pädiatrie in die Erwachsenenmedizin	050-003	S3	Angemeldet	N. z.

*AWMF* Arbeitsgemeinschaft der Wissenschaftlichen Medizinischen Fachgesellschaften; *DGKJP* Deutsche Gesellschaft für Kinder- und Jugendpsychiatrie, Psychosomatik und Psychotherapie e. V.; *DGPPN* Deutsche Gesellschaft für Psychiatrie, Psychotherapie, Psychosomatik und Nervenheilkunde e. V.; *N. z.* Nicht zutreffend; *S1* Handlungsempfehlungen von Expertinnen und Experten, *S2k* konsensbasiert, *S2e* evidenzbasiert, *S3* evidenz- und konsensbasiert

## Notwendigkeit einer evidenzbasierten Suizidprävention in Deutschland

Zwischen 1980 und 2020 kam es zu einer signifikanten Abnahme von Suiziden in Deutschland um mehr als 40 % [1], mutmaßlich unter anderem als Folge einer verbesserten psychiatrisch-psychotherapeutischen Versorgung, eines Ausbaus der niedrigschwelligen Suizidpräventionsangebote (z. B. Krisendienste) und möglicherweise auch von Antistigmabemühungen und konzertierten Aktionen (beispielsweise „Deutsches Bündnis gegen Depression“; [36]). Seither stagnieren die Todesraten durch Suizid auf einem Niveau von ca. 12/100.000 Einwohner. Allerdings ist in den letzten 20 Jahren die Zahl der Todesfälle durch „sonstige oder nicht näher bezeichnete Todesursachen“ angestiegen, worunter sich auch Suizide verbergen können. Möglicherweise könnten in Zukunft aufgrund der Einführung der Datenschutzgrundverordnung vom Statistischen Bundesamt vermehrt Fälle in diese Kategorie eingeordnet werden. Da es sich bei Suizid meist um einen prinzipiell verhinderbaren Tod handelt, sind intensivere Maßnahmen zur Suizidprävention indiziert. Neben dem individuellen Leiden liegt die hohe gesundheitsökonomische Relevanz des Themas auf der Hand. Die Folgekosten für Gesellschaft und Gesundheitssystem sind erheblich [37–39].

Aufgrund der Komplexität des Phänomens Suizidalität ist Suizidprävention eine vielschichtige Aufgabe: Im Bereich des Gesundheitswesens resultiert daraus ein komplexer Hilfebedarf bei der Koordination der unterschiedlichen ambulanten und stationären patientenzentrierten Behandlungsleistungen. Insbesondere aufgrund der Tatsache, dass zumindest teilweise und vorübergehend Behandlungsmaßnahmen gegen den momentanen Willen der Patientinnen und Patienten eingeleitet werden müssen, ist eine Leitlinie für den Umgang mit Suizidalität im Erwachsenenalter auf höchstem Evidenzniveau dringend notwendig. In solchen Situationen muss die Leitlinie auch über den rein medizinischen Sektor hinausgehen und hat somit eine Relevanz für andere Berufsgruppen wie Einsatzkräfte, gesetzliche und psychosoziale Betreuerinnen und Betreuer, in der Seelsorge Tätige und andere. Eine Übertragung der Inhalte, insbesondere der Empfehlungen der S2k-Leitlinie „Suizidalität im Kindes- und Jugendalter“ [3], ist aufgrund altersspezifischer Unterschiede nur eingeschränkt möglich. Dies gilt auch für die damit verbundenen Anforderungen an die Hilfesysteme.

Aktuell werden von Kliniken häufig keine standardisierten Verfahren zum Umgang mit dem Thema „Suizidalität“ verwendet. Einzelne Kliniken setzen selbstentwickelte Standards und Verfahrensanweisungen zum Umgang mit Suizidalität ein. Diesen Empfehlungen und Standards liegt jedoch nicht immer wissenschaftliche Evidenz zugrunde und aus methodischen Gründen handelt es sich nicht um einen Konsens von Fachexpertinnen und -experten unter Einbeziehung von Betroffenen oder Angehörigen. Daraus ergibt sich unmittelbar die Notwendigkeit einer allgemeingültigen, wissenschaftlich fundierten Leitlinie für den Umgang mit Suizidalität in den unterschiedlichen ambulanten wie klinischen Versorgungsfeldern zur Verbesserung der Versorgungsqualität. Eine dezidierte Leitlinie zum Umgang mit suizidalem Verhalten im Erwachsenenalter gibt den beteiligten Akteurinnen und Akteuren der verschiedenen Sektoren eine klare Handlungsanweisung auf Grundlage der vorhandenen Evidenz und verbessert somit die Versorgung von ca. 200.000 von Suizidalität Betroffenen in Deutschland direkt.

Der Nachsorge und der Schnittstelle zum ambulanten Bereich bei Entlassung kommt ebenfalls eine besondere Rolle zu, da die Zeit direkt nach der Entlassung aus der stationären Behandlung eine Zeit des hohen Suizidrisikos ist [40, 41]. Klare Angaben zum möglichen Vorgehen bei der Vernetzung und Koordination der verschiedenen Akteure sind ein bedeutsamer Faktor. Durch eine entsprechende Leitlinie kann sowohl die interdisziplinäre (z. B. zwischen Notaufnahme und Psychiatrie oder im Konsiliar- und Liaisondienst), die intersektorale (z. B. zwischen ambulantem und stationärem Sektor) als auch die Koordination zwischen verschiedenen Berufsgruppen (z. B. dem medizinischen, psychologischen, pflegerischen und sozialarbeiterischen Fachpersonal) besser sichergestellt werden. Durch Konsentierung einer S3-Leitlinie besteht somit ein erhebliches Potenzial für die Verbesserung der Versorgung von betroffenen Patientinnen und Patienten (mit entsprechender Senkung von Mortalität und Morbidität) sowie von deren Angehörigen, auch im Rahmen der Postvention [42].

Auf Basis der beschriebenen Notwendigkeit nationaler Suizidpräventionsprogramme [43] sind die Darstellung und fortlaufende Aktualisierung der vorhandenen wissenschaftlichen Evidenz durch eine Leitlinie ein zentraler Baustein in der Bewertung der möglichen Maßnahmen. Erfreulicherweise wurde Ende August 2021 durch den Innovationsfonds des Gemeinsamen Bundesausschusses (G‑BA) die Finanzierung einer S3-Leitlinie „Umgang mit Suizidalität“ bewilligt. Diese Leitlinie ist bei der AWMF angemeldet (Tab. 1).

Wirkungsvolle Suizidprävention schließt auch Interventionen auf weiteren Handlungsebenen ein: Dazu gehören unter anderem eine umfängliche Erfassung von Daten zu Suizidversuchen und vollendeten Suiziden, die Enttabuisierung der Suizidthematik, eine verantwortungsvolle Berichterstattung über Suizide in den Medien und niedrigschwellige Angebote für Suizidgefährdete sowie eine datengeleitete Methodenrestriktion. Suizidprävention zielt auf die Vermeidung und Reduzierung von Suiziden, auf Hilfen für suizidale Personen wie auch auf die Hilfen für Hinterbliebene, die nach einem Suizid ein erhöhtes Risiko für psychische Erkrankungen haben und oft selbst Hilfe und Unterstützung benötigen [33]. Die Verfügbarkeit von Leitlinien ist dabei einer von vielen wichtigen Bausteinen in der Suizidprävention. Die Einrichtung einer unabhängigen zentralen Einrichtung für Suizidprävention zur bundesweiten Datenanalyse, von zentralen Beratungs- und Notfallangeboten (wie beispielsweise einer bundeseinheitlichen Notfallhotline) sowie Öffentlichkeitsarbeit und Multiplikatorschulung sind ebenfalls dringend geboten.

## Fazit

Bisher gibt es im deutschsprachigen Raum zwar eine S2k-Leitlinie zur Suizidalität im Kindes- und Jugendalter [3], jedoch keine Leitlinie zum Umgang mit Suizidalität und zur Suizidprävention im Erwachsenenalter. Die Fertigstellung einer S3-Leitlinie zum Umgang mit Suizidalität im Erwachsenenalter wird jedoch für 2024 erwartet. In einer S3-Leitlinie können Handlungsempfehlungen für die in den verschiedenen Sektoren des Gesundheitssystems Tätigen formuliert werden. Leitlinien zur Suizidalität und zur Suizidprävention tragen erheblich zu einer besseren Wissensvermittlung sowie zur Implementation vorhandener Evidenz in der Versorgung von Suizidgefährdeten und betroffenen Angehörigen bei, insbesondere auch in den nichtpsychiatrisch-psychotherapeutischen Fachgebieten. Basierend auf der wissenschaftlichen Evidenz, einem interdisziplinären Konsens zur Prävention suizidalen Verhaltens und der klinischen Behandlung suizidgefährdeter Personen können Standards für die Früherkennung, Klassifikation, Diagnostik, Therapie und Nachsorge geschaffen werden.

Bei Verdacht auf das Vorliegen einer psychischen Störung kann/sollte in der Leitlinie „Umgang mit Suizidalität“ auf die Leitlinien der jeweiligen psychischen Erkrankung bzw. Störung hingewiesen werden. Damit wird die individuelle, direkte Versorgung der betroffenen Patientinnen und Patienten optimiert und die Struktur- und Prozessqualität der mit Suizidalität befassten Einrichtungen des Gesundheitssystems verbessert, einschließlich an deren Schnittstellen zwischen ambulanter und stationärer Versorgung sowie zwischen einzelnen medizinischen Fachgebieten. Ein standardisiertes Vorgehen im Hinblick auf Erfassung von und Umgang mit Suizidalität ist daher ein wesentlicher Beitrag zur Patientensicherheit. Die Erstellung einer Patientenleitlinie, mithilfe derer sich sowohl Betroffene als auch Angehörige bzw. Hinterbliebene informieren können, ist darüber hinaus geplant.

## Footnotes

1 (https://app.magicapp.org/).
